# Basilar artery flow velocities and optic nerve sheath diameter as adjuvant tools for early diagnosis of hypoxic ischemic encephalopathy in neonates

**DOI:** 10.1186/s13052-026-02243-4

**Published:** 2026-04-10

**Authors:** Marwa Mohamed Farag, Mohamed Alaa Eldin Hassan Thabet, Islam SH Ahmed, Husam Mohamed Abu Halawa

**Affiliations:** 1 Pediatric Department, Alexandria University, Alexandria University Hospital, Alexandria, Egypt; 2 Ophthalmology Department, Alexandria University Hospital, Alexandria University, Alexandria, Egypt

**Keywords:** ONSD in neonate, Basilar artery Doppler, Perinatal asphyxia

## Abstract

**Objective:**

We investigated potentiality ultrasonography measured optic nerve sheath diameter (ONSD) and basilar artery (BA) velocities to identify neonates with HIE-requiring-TH.

**Methods:**

Study was of a case-control-design. Thirty- five neonates with mild-to-severe HIE admitted to NICU of Alexandria-University-Maternity-Hospital and were monitored by ultrasound-measured ONSD and BA velocities, in first-12-hours, during TH, and after rewarming. First scan measures of patients were compared to healthy-control-neonates. Comparative statistics, ROC-curves, and correlation of different clinical and imaging parameters were used in analysis.

**Results:**

Mean value of left-and-right-ONSD measurements in control group (0.31 cm, 0.30 cm, respectively) were significantly higher than asphyxiated-group (0.35 cm), with *p*-value<0.001. ONSDs of > 0.32 cm in right-and-left eyes carry 91% and 88% sensitivity, respectively, and 100% specificity in identifying patients with mild-to-severe HIE. Resistive index (RI) of BA decreased and EDV of BA increased in HIE-patients than healthy-control-neonates, with p value<0.001. Left-and-right-ONSD diameters are negatively correlated with BA-RI, with *r*=-.432; *p* < .01and *r*=-.34; *P*.046, respectively. ONSDs in both eyes, BA-RI and BA-EDV showed significant changes throughout three time points in HIE-patients.

**Conclusion:**

ONSD and BA-velocities can help in early identification of asphyxiated-neonates and therefore can be used to select patients candidate for TH. They also help in monitoring of asphyxiated-patient before, during and after TH.

**Supplementary Information:**

The online version contains supplementary material available at 10.1186/s13052-026-02243-4.

## Impacts

1. The current clinical criteria for diagnosing hypoxic ischemic encephalopathy in infants might overlook some patients, especially in low- and middle-income nations.

2. Basilar artery (BA) Doppler velocimetry and ultrasound measured optic nerve sheath diameter (ONSD) can be parts of point of care ultrasound in asphyxiated neonates.

3. ONSD, which is a reflection of intracranial pressure and BA velocimetry, which is a reflection to cerebral blood flow might help in identification of patients candidate for therapeutic hypothermia (TH) and monitoring of asphyxiated neonates undergoing TH.

**Supplementary Information:**

The online version contains supplementary material available at 10.1186/s13052-026-02243-4.

## Background

Neonatal hypoxic ischemic encephalopathy (HIE) is a devastating disease that primarily causes neuronal injury and a predominant contributor of neonatal death and disability globally [1]. Incidence of HIE in low- middle income countries (LMIC) is relatively high [2]. This might be due to lack of antenatal care, high incidence of home deliveries, inadequate intrapartal monitoring and deliveries being unattended by experienced staff. The strict criteria for diagnosing HIE has been developed by AAP-ACOG and adopted since then. The problem of those criteria that it was designed to focus on most severely asphyxiated infants and may miss out some cases of HIE due to factors such as milder hypoxia induced acidosis or higher Apgar scores; missed laboratory investigations; uncommon patterns of brain injury on MRI; unavailability of MRI; incomplete data about the outcome that miss milder motor deficit; or insufficient data gathering [3]. 

In LMIC, the problem can be exaggerated by inadequate resuscitation, and unavailability of cord blood gas and/or blood gas in the first hour of life, subjectivity of clinical criteria unavailability of aEEG. Therefore, authors to investigate markers that might be added regionally to this criterion or might help the clinician to identify asphyxiated neonates lacking full criteria of AAP/ACOG that might require TH. Those markers might be laboratory like AST ALT, CK-mb and Troponin or monitoring tools like cerebral oxygenation measures [4–6]. 

Ultrasound is bedside, less-costly than MRI, if already available in hospital, that might also help in solving this clinical dilemma. In addition, it might help in identification of asphyxiated patients candidate for expensive MRI in LMIC. The ONSD as indicator of pathophysiological changes in hypoxiated brain that can be easily applied and is an attractive tool for neonatologist to help in identification of patients with HIE candidate for TH. ONDS has been extensively studied as indicator of increased intracranial pressure (ICP) in adult and pediatrics [7]. However, few studies and data were published about its normatives and uses in neonates [8, 9]. In addition, hypoxia induces changes in cerebral blood flow (CBF) of brain that can be measured using cerebral Doppler velocimetry.

The aim of the present study is to investigate the utility of ONSD and basilar artery velocities in identification and monitoring of neonates with HIE candidate for therapeutic hypothermia (TH).

## Methods

This prospective study was conducted in neonatal intensive care unit (NICU) of Alexandria University Maternity Hospital between begin December 2023 till end of July 2024. All asphyxiated neonates with HIE between gestational age of ≥ 37weeks (*n* = 47), were selected consecutively and planned for TH with respect to the criteria of The American Academy of Pediatrics (AAP) and the American College of Obstetricians and Gynecologists (ACOG) of birth asphyxia [10]. Patients with critical congenital heart diseases (*n* = 2), severe cardiovascular instability and/or severe PPHN and TH were discontinued before 72 h (*n* = 3) or not initiated (*n* = 2), patient with intracranial hemorrhage (*n* = 1), and patients with incomplete scan due to unavailability of device (*n* = 4) were excluded from the analysis.

All patients in asphyxiated-group were fulfilling the eligibility criteria for TH and subjected to whole body cooling, passive and active, using cold gel packs (*n* = 27), or Blanketrol III (*n* = 8), according to availability of device. Blanketrol III device (GENTHERM Company, serial No: 106130311862072 [21] 202311419) is preferentially used for lower gestational age of ≤ 37 weeks and birth weight of ≤ 2.7 kg in which temperature is difficult to be maintained stable using passive cooling +/- non-freezed cold gel packs, (supplement 1).

Written informed parental consent was obtained from parent. The study was approved by the ethics committee of the Alexandria University with ID number 0107702, IRB number 00012098, and FWA number 00018699.

Ultrasonographic scans (transcranial Doppler and ocular measurements): Scans were done in the first 12 h after birth, during TH and after termination of TH. A single operator who was trained for 2 years in transcranial sonography and ONSD measurement, performed all scans to be assessed by consultant neonatologist, experienced in point of care ultrasound reviewed images. A mobile device model (Philips HD11 XE, USA) was used, with L12-3 probe with a frequency range of 12–3 MHz (for ocular measurements) and s8-3 probe with a frequency range of 8 − 3 MHz (for transcranial Doppler).

**Fig. 1:**
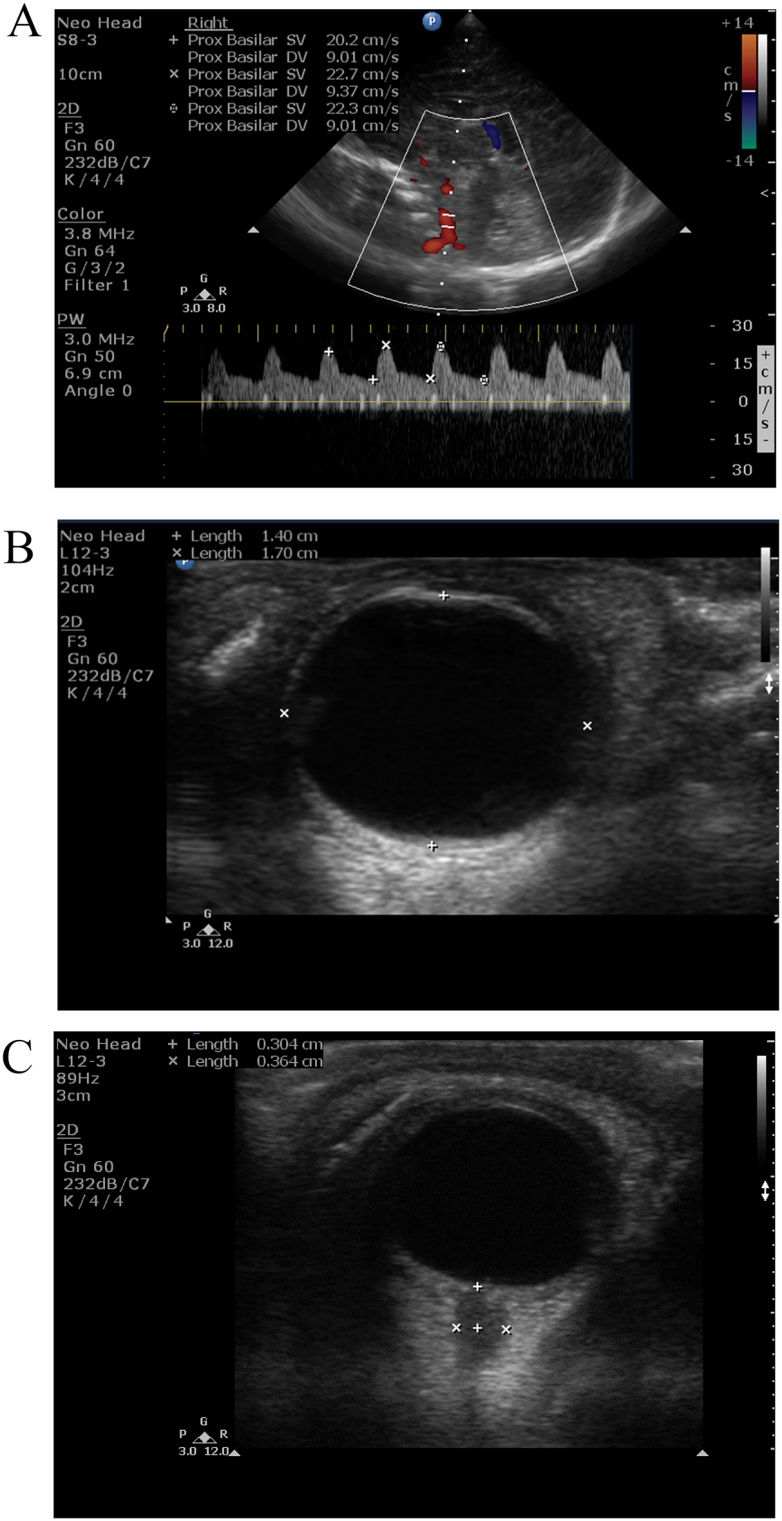
RI of BA in patient with severe HIE within first 12 hours after birth, with low RI indicating altered cerebral perfusion in HIE and a reduction in cerebrovascular resistance (**A**). ocular sonography showed ocular dimensions (**B**) and large optic nerve sheath diameter (**C**)

Peak systolic velocity (PSV), end diastolic velocity (EDV) and resistive index (RI) of the BA Resistive index (RI) was calculated as the peak systolic velocity minus the end diastolic velocity divided by the systolic velocity. RI was calculated from PSV and EDV and averaged from 3 measures. ONSD was measured through transorbital window within 24 h after birth, and during and after termination of TH. Measures of BA velocimetry were averaged from 3 measures, Fig. 1. ONSD was measured bilaterally 3 mm behind the orbit. The point of measurement is 3 mm away from the fovea posteriorly.

Sample size: Based on the study of Pishdad et al. [11], a minimal required sample size of 60 neonates is needed to assess the role of using doppler sonography resistive index of basilar artery for the diagnosis of perinatal asphyxia that detect difference of 0.2 between the null hypothesis AUC of 0.5 and the alternative hypothesis AUC of 0.7, using One ROC Curves Power Analysis which achieves 80% power with a target significance level at 5%. Sample size was calculated using NCSS 2004 and PASS 2000 program.

Data were fed to the computer and analyzed using IBM SPSS software package version 20.0. The Kolmogorov–Smirnov test was used to verify the normality of distribution. Qualitative data were described using number and percent. Quantitative data were described using range (minimum and maximum), mean, and standard deviation, median and interquartile range (IQR). Significance of the obtained results was judged at the 5% level. Student t-test, Monte Carlo test, Chi-square test, Mann Whitney U test and Fisher Exact test were used for comparison between the two groups and the asphyxiated group at the three time points regarding different variables. Receiver operating curve (ROC) was constructed to test sensitivity and specificity of ONSD and BA velocities in early identification of hypoxia. Pearson and Spearman correlation test were used to detect relationships between different variables.

## Results

Table 1 shows comparison Meconium staining, and maternal risk factors were higher in asphyxiated group. Apgar scores were significantly lower in the asphyxiated group across all time points (1, 5, and 10 min) compared to the controls. Additionally, heart rate was significantly lower in asphyxiated group.

**Table 1:** Comparing between asphyxiated and control groups regarding demographic features and clinical history

	Study groups				
	**asphyxiated group** **(No.=35)**		**Control group** **(No.==35)**		**Test of significance** **(p)**
	***No.***	***%***	***No.***	***%***	
**Sex**					(X^2^ = 3.05, *P*=.08)
• Male	26	74.3	19	54.3	
• Female	9	25.7	16	45.7	
**Mode of delivery**					(X^2^ = 14, *P*<.001*)
• Normal vaginal delivery (NVD)	20	57.1	5	14.3	
• Cesarean section (CS)	15	42.9	30	85.7	
**Mode of anesthesia**					(X2 = 1.56, *P*=.21)
• Spinal	20	57.1	4	11.4	
• General	11	31.4	31	88.6	
• No	4	11.4	0	0.0	
**Previous sibling death**					-
• Yes	0	0.0	0	0.0	
• No	35	100.0	35	100.0	
**Meconium stained**		0.0			(X2 = 14.5, *P*<.001*)
• Vigorous	2	5.7	0	0.0	
• Flaccid	10	28.6	0	0.0	
• No	23	65.7	35	100.0	
**Sentinal event**					MCP<0.001*
• No	17	48.6	35	100.0	
• Accidental hge	3	8.6	0	0.0	
• Prom	2	5.7	0	0.0	
• Obstructed labour	10	28.6	0	0.0	
• > Events	3	8.6	0	0.0	
**Maternal medical history**					
• Negative	14	40.0	35	100.0	MCP<0.001*
• Anemia	3	8.6	0	0.0	
• Infection	7	20.0	0	0.0	
• DM	1	2.9	0	0.0	
• PET	1	2.9	0	0.0	
• GHT	3	8.6	0	0.0	
• > Diseases	6	17.1	0	0.0	
**Resuscitations data**					MCP<0.001*
• Initial steps	6	17.1	26	74.3	
• PPV	22	62.9	9	25.7	
• ETT	7	20.0	0	0.0	
**Gestational age(weeks)**					(U = 616,*P*=.96)
Mean ± SD	38.6 ± 1.4		38.45±0.81		
Median (Min-Max)	38(37–41)		38(37–41)		
**Birth weight (Kg)**					(U = 526.5,*P*=.31)
Mean ± SD	3.1±0.54		3.1±0.28		
Median (Min-Max)	2.9(2.5–4.6)		3.1(2.5–3.5)		
**Maternal age (Years)**					(t = 2.5,*P*=.014*)
Mean ± SD	29.4 ± 6.8		25.9 ± 4.3		
Median (Min-Max)	28(20–43)		26(19–37)		
**1-minute Apgar scores**					(U = 0,*P*<.001*)
Mean ± SD	3±0.8		6±0.6		
Median (Min-Max)	3(1–4)		6(5–7)		
**5-minute Apgar scores**					(U = 0,*P*<.001*)
Mean ± SD	5±0.95		8±0.5		
Median (Min-Max)	5(3–7)		8(8–9)		
**10-minute Apgar scores**					(U = 0, *P*<.001*)
Mean ± SD	7±0.8		9±0.23		
Median (Min-Max)	7(5–8)		9(9–10)		
**At the time of the scan**					
**HR(Beat/Minute)**					(U = 0, *P*<.001*)
Mean ± SD	96.2 ± 12.3		136.4 ± 7.9		
Median (Min-Max)	100(32–110)		135(125–150)		
**RR (Breath/Minute)**					(t = − 0.11, *P*=.9)
Mean ± SD	40 ± 7.1		40.34 ± 5.96		
Median (Min-Max)	40(28–55)		40(29–52)		
**BP (mm Hg)**					(U = 666.5, *P*=.52)
Mean ± SD	45 ± 5.38		44 ± 4.5		
Median (Min-Max)	45(37–55)		44(37–52)		
**CRT (sec)**					-
Median (Min-Max)	2(2–2)		2(2–2)		

**X**^**2**^; Pearson Chi-Square test MCP; Monte Carlo test *; Significant (*p* < .05)

U; MannWhitney test t; Independent t test, ETT endotracheal tube, PPV positive pressure ventilation, PET pre-eclamptic toxemia, DM diabetes mellitus, GHT gestational hypertension, HR heart rate, CRT capillary refill time, BP blood pressure, RR respiratory rate

**Table 2:** Comparison between the two study groups regarding ocular dimention, optic nerve sheath diameter and Doppler velocimetry

Parameters	Side	Healthy Controls group (No.=35)	Asphyxiated group (No.==35)			Test of significance (P1)	Test of significance (P2)
			Time of scans				
		1st 24 h	1st 24 h	1st 48 h	After re warming		
ONSD (cm)	Lt ^A^	0.31(0.3-0.32)	0.35(0.3.39)	0.32(0.29-0.36)	0.30(0.29-0.32)	(U = 44.5,*P*<.001*)	(X^2^_Friedman test_ =67.1, *P*<.001*)
	Rt ^B^	0.30(0.3-0.32)	0.35(0.3-0.37)	0.33(0.30-0.35)	0.30(0.29-0.32)	(U = 7.5,*P*<.001*)	(X^2^_Friedman test_ =67.04, *P*<.001*)
Ocular Vertical Diameter(cm)	Lt	1.45(1.40–1.50)	1.45(1.35–1.50)	1.45(1.35–1.50)	1.45(1.35–1.50)	(U = 481.5,*P*=.1)	(X^2^_Friedman test_ =0, *P* = 1)
	Rt	1.45(1.40–1.50)	1.45(1.35–1.50)	1.45(1.35–1.50)	1.45(1.35–1.50)	(U=,536.5P=0.34)	(X^2^_Friedman test_ =0, *P* = 1)
Ocular Horizontal Diameter (cm)	Lt	1.70(1.65–1.75)	1.70(1.65–1.80)	1.70(1.65–1.80)	1.70(1.65–1.80)	(U = 580.5,*P*=.65)	(X^2^_Friedman test_ =4, *P*=.135)
	Rt	1.70(1.60–1.75)	1.70(1.65–1.80)	1.70(1.65–1.80)	1.70(1.65–1.80)	(U = 581,*P*=.6)	(X^2^_Friedman test_ =0, *P* = 1)
Ocular Diameter Ratio (Ocular Vertical Diameter / Ocular Horizontal Diameter) (%)	Lt	85.29(82.35–88.24)	84.84(77.78–88.24)	84.84(77.78–88.24)	85.3(77.78–100)	(U = 460,*P*=.07)	(X^2^_Friedman test_ =4, *P*=.135)
	Rt	85.7(80-88.24)	84.29(77.78–88.24)	84.29(77.78–88.24)	84.29(77.78–88.24)	(U = 466,*P*=.08)	(X^2^_Friedman test_ =0, *P* = 1)
PSV (m/sec) of BA ^C^		33.3(14–54)	32.50(18.30–62.00)	33(18.30–57.60)	38.5(7.20–61)	(U = 589,*P*=.78)	(X^2^_Friedman test_ =14.2, *P*=.001*)
EDV (m/sec) of BA ^D^		9.92(4–16)	12.5(5-26.10)	11.5(6.60–24.80)	11.5(7.80–19.50)	(U = 942.5,*P*<.001*)	(X^2^_Friedman test_ =7.35, *P*=.025*)
RI of BA^E^		0.71(0.68-0.84)	0.59(0.52-0.87)	0.66(0.55-0.69)	0.71(0.64-0.76)	(U = 35,*P*<.001*)	(X^2^_Friedman test_ =62.4, *P*<.001*)

Data described Median (Min-Max)

P1; Sig betw. asphyxiated group (Cases) at1st 24 h Vs controls at1st 24 h

P2; Sig bet. Time episodes of asphyxiated group (cases)

U; MannWhitney test

*X*^*2*^_*Friedman test*_; Friedman test

A; Pairwise comparison Sig bet. [After warming Vs (1st 24 h&1st 48 h) ](*P*<.001,0.002 respectively), [(1st 24 h&1st 48 h)as *p*<.001)

B; Pairwise comparison Sig bet. [After warming Vs (1st 24 h&1st 48 h) ](*P*<.001,.<0.001 respectively), [(1st 24 h&1st 48 h)as *p*<.001)

C; Pairwise comparison Sig bet. [After warming Vs (1st 24 h&1st 48 h) ](*P*=.005,.=0.003 respectively)

D; Pairwise comparison Sig bet. (1st 24 h&1st 48 h)as *p*=.026)

E; Pairwise comparison Sig bet. [After warming Vs (1st 24 h&1st 48 h) ](*P*<.001,.<0.001 respectively), [(1st 24 h&1st 48 h)as *p*=.001)

**Table 3:** Correlation between different biomarkers in the first 24 h with measures (optic n diameter, orbital diameters, PSV, EDV and RI) in right and left eyes

right		RT ONSD	RT Ocular vertical diameter	RT Ocular horizontal diameter	RT Ocular diameter ratio	PSV of BA	EDV of BA	RI of BA	HR (Beat/minute)	Creatinine (mg/dl)	Troponin (ng/ml)	Platelets (10^3/ul)	AST (U/L)	ALT (U/L)	Initial PH	Initial BE	Ck-MB
RT ONSD	r	**1**	− 0.010	0.097	− 0.060	0.055	− 0.016	− 0.340	− 0.398	0.203	0.098	− 0.163	0.013	0.075	− 0.436	− 0.289	0.437
	p		0.956	0.578	0.730	0.755	0.929	.046*	0.018*	0.243	0.577	0.350	0.942	0.666	0.009*	0.092	0.012*
RT Ocular Vertical Diameter	r	− 0.010	1	0.213	0.924	0.119	0.022	0.212	0.110	0.028	− 0.082	0.181	0.001	0.183	− 0.160	− 0.293	− 0.155
	p	0.956		0.219	< 0.001*	0.496	0.898	0.222	0.528	0.875	0.639	0.298	0.996	0.293	0.360	0.087	0.397
RT Ocular Horizontal Diameter	r	0.097	0.213	1	− 0.162	0.013	0.058	0.068	0.030	0.017	− 0.079	0.275	0.091	0.198	− 0.011	− 0.087	− 0.125
	p	0.578	0.219		0.352	0.939	0.740	0.698	0.866	0.921	0.653	0.109	0.601	0.255	0.949	0.619	0.496
RT Ocular Diameter Ratio	r	− 0.06	0.924	− 0.162	1	0.087	− 0.017	0.182	0.093	0.024	− 0.068	0.081	− 0.037	0.092	− 0.154	− 0.268	− 0.132
	p	0.730	< 0.001*	0.352		0.620	0.924	0.296	0.597	0.892	0.698	0.643	0.833	0.597	0.378	0.119	0.471
PSV of BA	r	0.055	0.119	0.013	0.087	1	0.892	− 0.224	− 0.201	− 0.010	0.171	− 0.079	0.216	0.111	0.005	− 0.152	0.312
	p	0.755	0.496	0.939	0.620		< 0.001*	0.195	0.246	0.955	0.325	0.651	0.213	0.527	0.977	0.384	0.082
EDV of BA	r	− 0.016	0.022	0.058	− 0.017	0.892	1	− 0.424	− 0.217	− 0.106	0.1	− 0.081	0.114	− 0.024	− 0.013	− 0.182	0.250
	p	0.929	0.898	0.740	0.924	< 0.001*		.011*	0.211	0.544	0.568	0.643	0.514	0.891	0.941	0.296	0.168
RI of BA	r	− 0.340	0.212	0.068	0.182	− 0.224	− 0.424	_**1**_	0.370	− 0.102	− 0.248	0.520	− 0.183	0.052	0.321	0.389	− 0.377
	p	0.046*	0.222	0.698	0.296	0.195	0.011*		0.029*	0.561	0.150	0.001*	0.293	0.768	0.060	0.021*	0.033*
Thompson score	r	0.504	0.166	0.033	0.142	0.235	0.190	− 0.646	− 0.384	0.281	0.548	− 0.376	0.351	0.323	− 0.537	− 0.637	0.558
	p	0.002*	0.342	0.850	0.417	0.174	0.273	0.001*	0.023*	0.102	0.001*	0.026*	0.039*	0.059	0.001*	0.001*	0.001*
Left		**LT ONSD**	**LT Ocular vertical diameter**	**LT Ocular horizontal diameter**	**LT Ocular diameter ratio**	**PSV of BA**	**EDV of BA**	**RI of BA**	**HR** **(Beat/minute)**	**Creatinine** **(mg/dl)**	**Troponin** **(ng/ml)**	**Platelets** **(10^3/ul)**	**AST** **(U/L)**	**ALT** **(U/L)**	**Initial PH**	**Initial BE**	**Ck-MB**
LT ONSD	r	**1**	0.130	0.243	0.050	0.085	0.073	− 0.432	− 0.390	0.265	0.098	− 0.170	0.153	0.207	− 0.557	− 0.441	0.452
	p		0.455	0.160	0.775	0.627	0.678	0.010*	0.021*	0.123	0.577	0.328	0.379	0.232	0.001*	0.008*	0.009*
LT Ocular Vertical Diameter	r	0.130	1	0.229	0.924	0.119	0.124	0.112	0.112	− 0.042	− 0.082	0.209	− 0.022	0.148	− 0.197	− 0.350	− 0.207
	p	0.455		0.187	0.001*	0.496	0.476	0.522	0.521	0.812	0.639	0.228	0.902	0.398	0.257	0.04*	0.256
LT Ocular Horizontal Diameter	r	0.243	0.229	1	− 0.150	− 0.029	0.027	− 0.068	0.191	0.247	− 0.007	0.260	0.252	0.336	− 0.086	− 0.201	0.019
	p	0.160	0.187	.	0.391	0.867	0.879	0.700	0.271	0.152	0.970	0.132	0.145	0.049*	0.622	0.246	0.920
LT Ocular Diameter Ratio	r	0.050	0.924	− 0.150	1	0.113	0.099	0.145	0.033	− 0.134	− 0.115	0.111	− 0.121	0.009	− 0.169	− 0.272	− 0.228
	p	0.775	0.001*	0.391	.	0.519	0.570	0.406	0.851	0.441	0.511	0.525	0.490	0.960	0.331	0.114	0.209
PSV of BA	r	0.085	0.119	− 0.029	0.113	1	0.892	− 0.224	− 0.201	− 0.010	0.171	− 0.079	0.216	0.111	0.005	− 0.152	0.312
	p	0.627	0.496	0.867	0.519	.	0.001*	0.195	0.246	0.955	0.325	0.651	0.213	0.527	0.977	0.384	0.082
EDV of BA	r	0.073	0.124	0.027	0.099	0.892	1	− 0.424	− 0.217	− 0.106	0.100	− 0.081	0.114	− 0.024	− 0.013	− 0.182	0.250
	p	0.678	0.476	0.879	0.570	0.001*		0.011*	0.211	0.544	0.568	0.643	0.514	0.891	0.941	0.296	0.168
RI of BA	r	− 0.432	0.112	− 0.068	0.145	− 0.224	− 0.424	1	0.370	− 0.102	− 0.248	0.520	− 0.183	0.052	0.321	0.389	− 0.377
	p	0.01*	0.522	0.700	0.406	0.195	0.011*		0.029*	0.561	0.150	0.001*	0.293	0.768	0.060	0.021*	0.033*
Thompson score	r	0.598	0.140	0.259	0.024	0.235	0.190	− 0.646	− 0.384	0.281	0.548	− 0.376	0.351	0.323	− 0.537	− 0.637	0.558
	p	.001*	0.421	0.133	0.892	0.174	0.273	0.001*	0.023*	0.102	0.001*	0.026*	0.039*	0.059	0.001*	0.001*	0.001*

r; Spearman correlation, RT right, LT left

The distribution of cases based on the Sarnat and Thompson scoring systems at the time of admission provides a clear picture of the severity of HIE in the study group. The majority of neonates (68.6%) presented with moderate HIE, with Thompson ranged between11to14. A smaller proportion of neonates were classified into severe HIE (22.9%, Thompson score = 15–22), while only 8.6% had mild HIE (Thompson score ranged between1-10) at admission. The correlation between Sarnat score and Thompson was previously reported by Bhagwani et al. [12] Supplement 2 demonstrates clinical course and laboratory biomarkers of asphyxiated group.

**Fig. 2:**
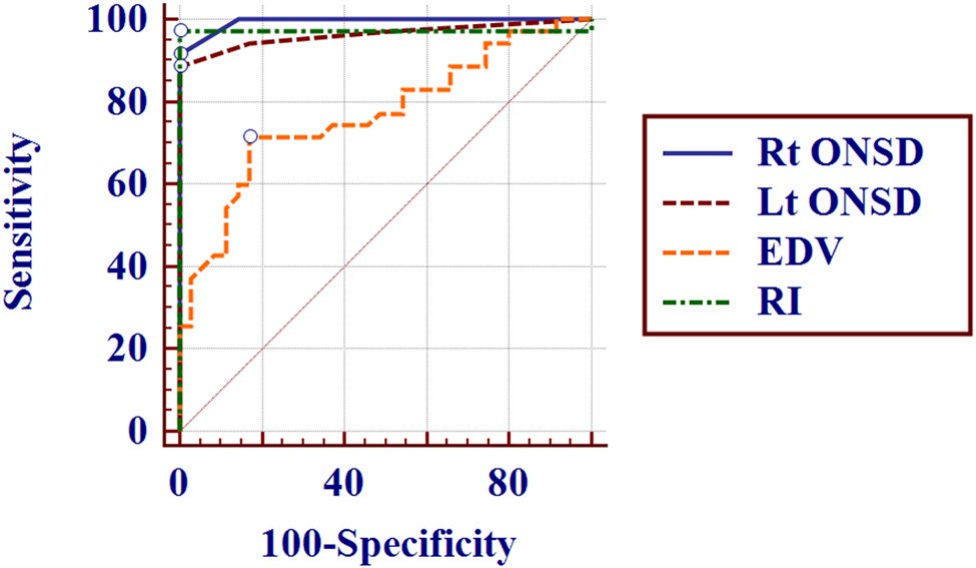
ROC curves for right ONSD, left ONSD, BA-EDV and BA-RI in diagnosis of hypoxic ischemic encephalopathy

The ROC curves of right and left ONSD at DOL1 (Fig. 2, supplement 3) depicted that a right or Lt ONSD > 0.32 cm were predictive for presence of HIE with sensitivity of 91.43% and 88.57%, respectively, and with similar specificity (100%). At DOL1, BA-RI ≤ 0.66 and BA EDV > 11.2 had sensitivity of 97.33% and 71.43%, with specificities of 100% and 80.6%, respectively, to predict HIE.

Table 2 demonstrates significant differences between control and asphyxiated groups regarding right and left ONSD, as well as cerebral Doppler velocities (BA-RI and BA-EDV), with p values < 0.001. In addition, it illustrates temporal changes in ONSD (right and left) and all measured BA velocities (PSV, EDV and RI) in both study groups, with p values < 0.001.

Table 3 showed the significant correlations between BA velocities, ONSD on both sides, ocular dimensions with clinical parameters like Thompson score and heart rate, and asphyxia biomarkers including Platelet count, creatinine, AST, ALT, Troponin I, CK-mb, PH and BD.

## Discussion

The current investigation elucidated the physiological differences in cerebral flow and ocular parameters between a control group and asphyxiated neonates underwent TH over different time intervals. This comparative analysis reveals significant differences in the ONSD, peak systolic velocity (PSV), end-diastolic velocity (EDV), and resistance index (RI) of the basilar artery (BA), which are critical indicators of intracranial pressure (ICP) and cerebral blood flow dynamics in neonatal HIE.

For our knowledge, Ceran et al’s research was the first published work describing ONSD in asphyxiated neonates compared to healthy control patients. However, they did not find a significant value of use of ONSD in asphyxiated neonate. Albeit our research identified ONSD as a useful non-invasive tool in monitoring asphyxiated patients before, during and after TH. The contradictory results might be due to different timing of measurements, as Ceran et al. measured ONSD in a later time, the first 24–48 h, at which cytotoxic oedema might have been resolved. Cytotoxic edema only occurs in the first few hours after the hypoxic insult and begin to resolve thereafter. In the current work we did the first scan in the first 12 h of life. Additionally, second and third scans were done at 48–72 h (during TH) and at 96–120 h (after rewarming). Serial measures indicates that ONSD decrease by time and pointing to the fact that cytotoxic brain edema resolves by time.

In the current study we used BA Doppler velocities. We speculate that BA might be important in monitoring of asphyxiated neonates because of the following reasons. Firstly, the only easily visualized unpaired artery suppling the whole brain rather than one cerebral hemisphere. It avoids the Dilemma of ACA flow measures in the sagittal plane where clinician never knows which artery is measured right or left. Secondly, it is straight artery with almost zero angle of insinuation when using Doppler cursor and also easily detected by the clinician. Thirdly, the main stem of BA has tiny pontine branches and no large branches arise from mid-stem that can alter velocities’ measures. However, there are two considerations, first being of small caliber, second it is not a branch or continuation of internal carotid artery which is main vessel supplying the cerebrum. Albeit the hemodynamic and CBF changes with asphyxia would reasonably affect all vessels of brain at various degrees.

The ONSD is a non-invasive surrogate marker for elevated ICP in asphyxiated neonates. In this study, ONSD measurements demonstrated a clear distinction between the asphyxiated group and the control group during the first 24 h, with significantly larger diameters observed in the asphyxiated group. The left ONSD measured a median of 0.35 mm in the asphyxiated group at 24 h, compared to 0.31 mm in the control group, with a highly significant p-value < 0.001. This enlargement of ONSD is consistent with increased ICP likely due to cerebral edema, a common consequence of HIE. However, after rewarming, the ONSD values in the asphyxiated group normalized to values comparable to the control group (Lt: 0.30 mm), suggesting that TH might have effectively reduced the intracranial pressure and cerebral edema in these neonates. The statistically significant differences observed between OND at the different time points within the asphyxiated group (*P* < .001) further support the dynamic changes in ICP before, during and after TH. Increased ICP occurs usually within the first few hours after a hypoxic-ischemic event due to early cytotoxic edema.

The ocular vertical and horizontal diameters, along with the ocular diameter ratio, did not show significant differences between the HIE and control groups in the first 24 h. This suggests that while ONSD can be sensitive to changes in intracranial pressure, the overall globe dimensions remain relatively stable after hypoxia and during/after TH. These findings increase the specificity of ONSD as a marker for cerebral edema over the neonatal ocular dimensions.

The study identified significant correlations between ONSD and systemic biomarkers such as CK-mb. In addition, significant correlations were noted between ONSD and hemodynamic parameters including BA-RI and HR. Another important correlation was observed between ONSD, and both base deficits and PH of initial venous blood gas, within first hour after birth, indicating the severity of hypoxic insult.

These findings might reflect hemodynamic and systemic responses to hypoxia in asphyxiated neonates, and their association with ONSD provides a more comprehensive understanding of the severity of hypoxic brain injury. Elevated CK-mb levels could indicate myocardial strain or injury, and decreased HR indicates that hypoxia selectively de-presses automaticity and conduction in the SA and AV nodes.

The cerebral flow velocities measurement became more expendably used in preterm for prediction of IVH and for monitoring of birth asphyxia [13, 14]. The analysis of cerebral blood flow parameters, including peak systolic velocity (PSV) and end-diastolic velocity (EDV) of the basilar artery (BA), revealed notable differences between the asphyxiated and control groups, particularly at the 24-hour mark. The median EDV was significantly higher in the asphyxiated group at 24 h (12.5 cm/s) compared to the control group (9.92 cm/s, U = 942.5, *P* < .001), indicating altered cerebral perfusion in HIE. The resistance index (RI) of the BA was notably lower in the asphyxiated group at 24 h (median RI = 0.59) compared to the controls (median RI = 0.71), with a significant p-value (U = 35, *P* < .001). This decrease in RI suggests a reduction in cerebrovascular resistance, which may be a compensatory mechanism to maintain cerebral perfusion in the face of hypoxic-ischemic injury. In addition, decreased RI in the first might be an indicator of start of reperfusion phase when the diastolic flow starts to increase.

*K. R. Natique et al.* conducted a prospective cohort study to explore the use of serial US as a tool for detecting cerebral blood flow velocity and resistance index (RI) in neonates with HIE. The study involved 60 neonates, all born at or after 36 weeks of gestation, with perinatal acidemia and encephalopathy, along with a control group. Serial Doppler recordings were taken within the first 24 h of life, with a mean RI ≤ 0.55 considered abnormal. Results indicated that lower mean RIs shortly after birth were associated with increasing severity of HIE, with abnormal RIs observed in 61% of mild, 76% of moderate, and 100% of severe HIE cases. The study concludes that Doppler measurements can serve as valuable bedside biomarkers for assessing the time and severity of neonatal HIE [15].

Post-rewarming, the cerebral hemodynamics showed a return towards the baseline, with RI values in the asphyxiated group returning to normal (0.71), comparable to the control group, indicating a potential normalization of cerebrovascular resistance following therapeutic hypothermia. The significant changes in PSV and EDV observed through pairwise comparisons (e.g., after rewarming vs. 24 and 48 h, *P* = .005 and *P* = .003 respectively) highlight the impact of therapeutic hypothermia on cerebral blood flow dynamics.

Similar to the right eye, ONSD in the left eye demonstrated a significant negative correlation with the RI of BA (*r* = -.432, *P* = .01), confirming the relationship between increased cerebrovascular resistance and reduced ONSD. Also, there is significant correlation with platelet count BD, and CKmb.

Both BA-RI and ONSD(right and left eyes) are significantly correlated with each others and each of them was correlated to Thompson acore.Those results enhance the utility of BA velocities ONSD as non-invasive monitoring tool s that can integrate both local (cerebral) and systemic physiological responses to hypoxic-ischemic injury.

## Limitations of study

Many limitations can be acknowledged in the current work. MRI were not done early for all patients. Long term follow up was not feasible. Only 12 patients were compliant to have MRI. Only 2 patients had abnormal MRI with 3T MRI. Another limitation that sample size was relatively small. However, one of the goals of this study is to select patients candidate for MRI in LMIC based of easier and less expensive tools, OSND and BA velocities. Another limitation is use of hot and cold jel packs and unavailability ogf Blanketrol device for all patients. However, it might be helpful for lower income countries where HIE is major health problem and the cooling devices are not available. 

In addition, we included patients with mild HIE, as well as moderate to severe HIE that are usually excluded from hypothermia protocols. Nevertheless, cooling of mild HIE is adopted in the NICU of AUMH for several reasons: firstly, there is increasing evidence that infants who have mild encephalopathy in the first 6 h of life may still have a high risk for brain injury [16–18]. A systemic review of twenty studies found that a significant proportion of infants with mild HIE (86/341 (25%) of infants) have abnormal outcome at follow up [19]. This finding contrasts the historical observations that infants with mild encephalopathy had normal neurodevelopmental outcomes [20].

Secondly, the time of the insult affects the clinical presentation as well as the clinical progression of neonatal encephalopathy. Although the timing of the insult is predominantly perinatal, it is not always clearly identified and could encompass antenatal as well as acute on chronic compound insults. Infants who had a severe antenatal event may recover by the time of birth at which time the stage of encephalopathy is perceived as mild. In contrast, an infant with a more acute insult can have only mild abnormalities on neurological examination in the first 6 h of age which can then evolve to moderate or severe abnormalities after the first day of life. In addition, possible confounding variables such as maternal sedation, anesthesia, or tocolytics (e.g. magnesium sulfate) may play a role in the accurate determination of the stage of HIE when the neurological examination is performed immediately after birth [21]. Thirdly, unavailability of aEEG that might help as an objective tool to identify moderate to severe HIE.

In conclusion, the study highlights the potential utility of ONSD and BA Doppler velocities measurements as non-invasive tools for early detection of cerebral edema and variations in cerebral blood flow, and consequently, early identification and monitoring of brain injury in neonates with HIE undergoing TH. The observed correlations between ONSD, cerebral hemodynamics, and systemic biomarkers provide a foundation for developing more comprehensive monitoring strategies and optimizing therapeutic interventions in this vulnerable patient population. This work provides cut off value for ONSD measures that might help the clinician to identify asphyxiated neonates requiring therapeutic hypothermia with less personal variation, especially in absence of aEEG.

## Supplementary Information

Below is the link to the electronic supplementary material.

Supplementary Material 1

Supplementary Material 2

Supplementary Material 3

## Data Availability

The datasets generated during and/or analyzed during the current study are available from the corresponding author on reasonable request. Statement.

## References

[1] Korf JM, McCullough LD, Caretti V. A narrative review on treatment strategies for neonatal hypoxic ischemic encephalopathy. Transl Pediatr. 2023;12(8):1552–1571PMID: 37692539; PMCID: PMC10485647. 10.21037/tp-23-253. Epub 2023 Aug 22. 10.21037/tp-23-253 PMC10485647 37692539

[2] Admasu FT, Melese BD, Amare TJ, Zewude EA, Denku CY, Dejenie TA. The magnitude of neonatal asphyxia and its associated factors among newborns in public hospitals of North Gondar Zone, Northwest Ethiopia: A cross-sectional study. PLoS ONE. 2022;17(3):e0264816. 10.1371/journal.pone.0264816. PMID: 35245309; PMCID: PMC8896710. 35245309 10.1371/journal.pone.0264816 PMC8896710

[3] Molloy EJ, Branagan A, Hurley T, et al. Neonatal encephalopathy and hypoxic–ischemic encephalopathy: moving from controversy to consensus definitions and subclassification. Pediatr Res. 2023;94:1860–3. 37573378 10.1038/s41390-023-02775-z PMC13366674

[4] Farag MM, Khedr AAEAE, Attia MH, Ghazal HAE. Role of Near-Infrared Spectroscopy in Monitoring the Clinical Course of Asphyxiated Neonates Treated with Hypothermia. Am J Perinatol. 2024;41(4):429–38. 10.1055/s-0041-1740513. Epub 2021 Dec 29. PMID: 34965589. 34965589 10.1055/s-0041-1740513

[5] Reham Wagdya NL, Abdel-Wahaba O, Farag M. Diagnostic and prognostic role of troponin i in neonates with critical duct-dependent congenital heart diseases. Alexandria J Pediatr. 2023;36(2):86–94.

[6] Farag MM, Hesham Abd EL-R, Ghazal. Alaa Ibrahim and Bahaa Hammad. Near-infrared spectroscopy measured cerebral oxygenation in full-term infants during transition: an observational study. Egypt Pediatr Association Gaz. 2022;70:53.

[7] Lin JJ, Chen AE, Lin EE, Hsia SH, Chiang MC, Lin KL. Point-of-care ultrasound of optic nerve sheath diameter to detect intracranial pressure in neurocritically ill children - A narrative review. Biomed J. 2020;43(3):231–9. 10.1016/j.bj.2020.04.006. Epub 2020 Apr 23. PMID: 32335329; PMCID: PMC7424084. 32335329 10.1016/j.bj.2020.04.006 PMC7424084

[8] Lan SY, Tai HL, Lin JJ, Lan FY, Tsai HY, Lin KL. Measurement of optic nerve sheath diameter by ultrasound in healthy term neonates. Pediatr Neonatol. 2021;62(6):591–7. 10.1016/j.pedneo.2021.05.021. Epub 2021 Jun 19. PMID: 34226155. 34226155 10.1016/j.pedneo.2021.05.021

[9] Ceran B, Kutman HGK, Beyoğlu R, Şimşek GK, Elbayiyev S, Canpolat FE. Diagnostic role of optic nerve sheath diameter and brain blood flow in neonates with hypoxic-ischemic encephalopathy. Childs Nerv Syst. 2023;39(2):425–433. 10.1007/s00381-022-05731-0. Epub 2022 Nov 3. PMID: 36323955. 10.1007/s00381-022-05731-0 36323955

[10] American College of Obstetricians and Gynecologists. Neonatal encephalopathy and neurologic outcome. The American College of Obstetricians and Gynecologists, American Academy of Pediatrics. 2nd ed. 2014.

[11] Pishdad P, Yarmahmoodi F, Eghbali T, Arasteh P, Razavi SM. Using Doppler sonography resistive index for the diagnosis of perinatal asphyxia: a multi-centered study. BMC Neurol. 2022;22(1):104. 35305562 10.1186/s12883-022-02624-2 PMC8934006

[12] Bhagwani DK, Sharma M, Dolker S, Kothapalli S. To Study the Correlation of Thompson Scoring in Predicting Early Neonatal Outcome in Post Asphyxiated Term Neonates. J Clin Diagn Res. 2016;10(11):SC16–9. 10.7860/JCDR/2016/22896.8882. Epub 2016 Nov 1. PMID: 28050462; PMCID: PMC5198415. 28050462 10.7860/JCDR/2016/22896.8882 PMC5198415

[13] Farag MM, Gouda MH, Almohsen AMA, Khalifa MA. Intraventricular hemorrhage prediction in premature neonates in the era of hemodynamics monitoring: a prospective cohort study. Eur J Pediatr. 2022;181(12):4067–77. 10.1007/s00431-022-04630-5. Epub 2022 Sep 28. PMID: 36171508; PMCID: PMC9649466. 36171508 10.1007/s00431-022-04630-5 PMC9649466

[14] Kirimi E, Tuncer O, Atas B, Sakarya ME, Ceylan A. Clinical value of color Doppler ultrasonography measurements of full-term newborns with perinatal asphyxia and hypoxic ischemic encephalopathy in the first 12 hours of life and long-term prognosis. Tohoku J Exp Med. 2002;197(1):27–33. 10.1620/tjem.197.27. PMID: 12180790. 10.1620/tjem.197.27 12180790

[15] Natique KR, Das Y, Maxey MN, Sepulveda P, Brown LS, Chalak LF. Early Use of Transcranial Doppler Ultrasonography to Stratify Neonatal Encephalopathy. Pediatr Neurol. 2021;124:33–9. 34509001 10.1016/j.pediatrneurol.2021.07.004

[16] Murray DM, O’Connor CM, Ryan CA, Korotchikova I, Boylan GB. Early EEG Grade and Outcome at 5 Years After Mild Neonatal Hypoxic Ischemic Encephalopathy. Pediatrics. 2016;138(4):e20160659. 27650049 10.1542/peds.2016-0659

[17] Walsh BH, Neil J, Morey J, Yang E, Silvera MV, Inder TE, et al. The frequency and severity of magnetic resonance imaging abnormalities in infants with mild neonatal encephalopathy. J Pediatr. 2017;187:26–33. e1. 28479101 10.1016/j.jpeds.2017.03.065 PMC5533615

[18] Lodygensky GA, Battin MR, Gunn AJ. Mild Neonatal Encephalopathy—How, When, and How Much to Treat? JAMA Pediatr. 2018;172(1):3–4. 29114743 10.1001/jamapediatrics.2017.3044

[19] Conway J, Walsh B, Boylan G, Murray D. Mild hypoxic ischaemic encephalopathy and long term neurodevelopmental outcome-A systematic review. Early Hum Dev. 2018;120:80–7. 29496329 10.1016/j.earlhumdev.2018.02.007

[20] Robertson CM, Finer NN, Grace MG. School performance of survivors of neonatal encephalopathy associated with birth asphyxia at term. J Pediatr. 1989;114(5):753–60. 2469789 10.1016/s0022-3476(89)80132-5

[21] Chalak L, Latremouille S, Mir I, Sánchez PJ, Sant’Anna G. A review of the conundrum of mild hypoxic-ischemic encephalopathy: Current challenges and moving forward. Early Hum Dev. 2018;120:88–94. 29506900 10.1016/j.earlhumdev.2018.02.008

