# Supplementary material for: Basilar artery flow velocities and optic nerve sheath diameter as adjuvant tools for early diagnosis of hypoxic ischemic encephalopathy in neonates
Source: Ital J Pediatr. 2026 Apr 10;52:55. doi: 10.1186/s13052-026-02243-4 (PMC13069802; doi:10.1186/s13052-026-02243-4)
Supplement: Supplementary file 1 — Supplementary Material 1 [file 13052_2026_2243_MOESM1_ESM.docx]

**Supplement 1**

Therapeutic hypothermia

Onset: - TH was applied within 6 hours.

Duration: - 72 hours

Eligible infants underwent whole-body cooling therapy to achieve and maintain a Core body temperature between 33.5°C and 34.5°C. (Core temperature is monitored continuously using a rectal temperature probe). We used passive cooling +/- cold jel packs for 72 hours plus 12 hours rewarming

Passive cooling technique which depends on

Turning off the incubator, room temperature to be kept between 20 to 25, Patients were kept naked and away from windows, s figure 1.

Active cooling

Cooled-ice packs (non-freezed with temperature 10).

Rewarming

Temperature is increased gradually 0.5/2hours in 12 hours after termination of Th. First, put on blankets and then turning on the incubator with increasing in air temperature gradually.

Note: temperature is continuously monitored by rectal probe temperature sensor. The probe is inserted 2 cm depth through the rectal opening.


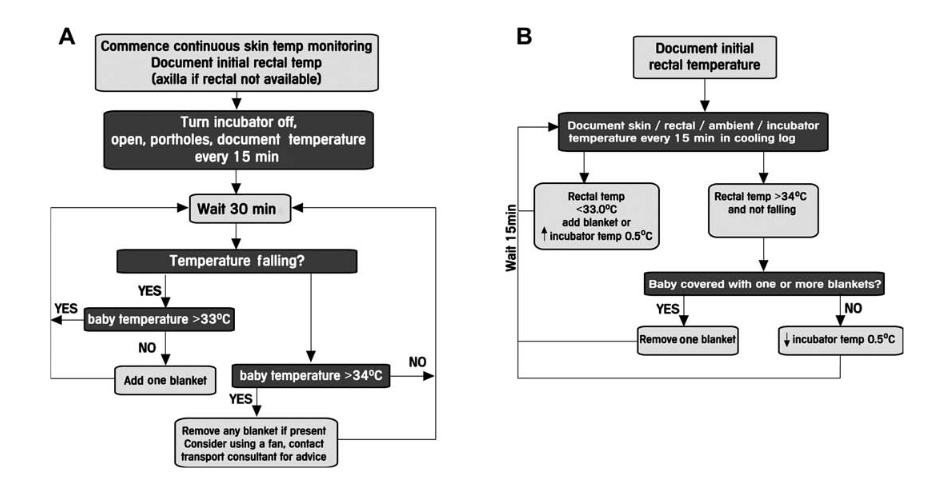


S-Figure1 : Passive cooling. (1)

1-Kendall GS, Kapetanakis A, Ratnavel N on behalf of the Cooling on Retrieval Study Group, et al. Passive cooling for initiation of therapeutic hypothermia in neonatal encephalopathy Archives of Disease in Childhood - Fetal and Neonatal Edition 2018;95:F408-F412.
