# Supplementary material for: Basilar artery flow velocities and optic nerve sheath diameter as adjuvant tools for early diagnosis of hypoxic ischemic encephalopathy in neonates
Source: Ital J Pediatr. 2026 Apr 10;52:55. doi: 10.1186/s13052-026-02243-4 (PMC13069802; doi:10.1186/s13052-026-02243-4)
Supplement: Supplementary file 2 — Supplementary Material 2 [file 13052_2026_2243_MOESM2_ESM.docx]

**Table (2): Distribution of cases of study as regards clinical presentations and laboratory biomarkers.**

| **Clinical presentations** | **Cases group**  **(No.=35)** | |
| --- | --- | --- |
|  |  |  |
|  | **No.** | **%** |
| **Sarnat at time of admission** |  |  |
| - Mild | 3 | 8.6 |
| - Moderate | 24 | 68.6 |
| - Severe | 8 | 22.9 |
| **Thompson at time of admission^(Ref)^** |  |  |
| - Score(1- 10) | 3 | 8.6 |
| - Score(11- 14) | 24 | 68.6 |
| - Score(15- 22) | 8 | 22.9 |
| **Thompson on discharge^(Ref)^** |  |  |
| - Score 0 | 8 | 22.9 |
| - Score(1- 10) | 27 | 77.1 |
| **Intractable Convulsions** |  |  |
| - Yes | 1 | 2.9 |
| - No | 34 | 97.1 |
| **Respiratory support** |  |  |
| - None | 23 | 65.7 |
| - NCPAP | 6 | 17.1 |
| - Minimal support | 6 | 17.1 |
| **Method of feeding on discharge** |  |  |
| - Oral | 34 | 97.1 |
| - Orogastric tube | 1 | 2.9 |
| **Fate (Discharged)** | 35 | 100 |
| **Time of initial feeding (Days)Median (Min-Max)** | 4 (4-4) | |
| **Age at starting hypothermia (Days)(Mean ± SD), Median (Min-Max)** | (3±1.1), 3(2-5) | |
| **Duration of hospital stay (Days) ((Mean ± SD), Median (Min-Max)** | (6±.87), 6(4-8) | |
|  | **Mean ± SD** | **Median (Min-Max)** |
| **Initial PH** | 7.18±.1 | 7.17(7-7.22) |
| **Initial HCO3( mmo/L )** | 10.45±3.3 | 10.5(3.1-15.3) |
| **Initial BE (mmol/L)** | -17.86±3.7 | -17.4(-28.2--12.1) |
| **Hb( g/dl)** | 16.01±2.3 | 16(11.5-20.3) |
| **WBC 10^3^/dl** | 16.31±5.6 | 15.6(4.2-29.9) |
| **Platelets 10^3^/dl** | 227.91±56.3 | 226(123-343) |
| **CRP Mg/L** | 3.33±2.3 | 2.7(0.05-9.7) |
| **AST IU/L** | 186.40±125.4 | 129(48-546) |
| **ALT IU/L** | 84.80±107.6 | 42(8-522) |
| **CK-MB U/L** | 91.42±76.5 | 63.6(17.2-320) |
| **TroponinI ng/ml** | 0.38±.44 | 0.2(0.05-2.05) |
| **Creatinine mg/dl** | .91±.31 | 0.84(0.3-1.49) |

NCPAP nasal continuous positive pressure
