# Supplementary material for: Basilar artery flow velocities and optic nerve sheath diameter as adjuvant tools for early diagnosis of hypoxic ischemic encephalopathy in neonates
Source: Ital J Pediatr. 2026 Apr 10;52:55. doi: 10.1186/s13052-026-02243-4 (PMC13069802; doi:10.1186/s13052-026-02243-4)
Supplement: Supplementary file 3 — Supplementary Material 3 [file 13052_2026_2243_MOESM3_ESM.docx]

**Supplement 2: Diagnostic abilities of ONSD and Ocular Diameter.**

| **NPV**  **(95%CI)** | **PPV**  **(95%CI)** | **Specificity**  **(95%CI)** | **Sensitivity**  **(95%CI)** | **cut of point** | **P value** | **AUC**  **(95%CI)** |  |
| --- | --- | --- | --- | --- | --- | --- | --- |
| 92.1 (78.6 - 98.3) | 100.0 89.1 - 100) | 100.00 (90.0 - 100.0) | 91.43 (76.9 - 98.2) | >0.32 | <.001* | .99(0.937 to 1) | **Rt ONSD** |
| 89.7 (75.8 - 97.1) | 100.0 (88.8 - 100.0) | 100.00 (90.0 - 100.0) | 88.57 (73.3 - 96.8) | >0.32 | <.001* | 0.964 (0.889 to 0.994) | **Lt ONSD** |
| 74.4 (57.9 - 87.0) | 80.6 (62.5 - 92.5) | 82.86 (66.4 - 93.4) | 71.43 (53.7 - 85.4) | >11.2 | <.001* | .769(0.653 to 0.862) | **EDV of BA** |
| 97.2( 85.5 - 99.9 ) | 100.0( 89.7 - 100.0) | 100.00 (90.0 - 100.0) | 97.14( 85.1 - 99.9) | ≤0.66 | <.001* | .971(0.901 to 0.997) | **RI of BA** |
